# Secondary Metabolites Produced during Aspergillus fumigatus and Pseudomonas aeruginosa Biofilm Formation

**DOI:** 10.1128/mbio.01850-22

**Published:** 2022-07-20

**Authors:** Rafael Wesley Bastos, Daniel Akiyama, Thaila Fernanda dos Reis, Ana Cristina Colabardini, Rafael Sanchez Luperini, Patrícia Alves de Castro, Regina Lúcia Baldini, Taícia Fill, Gustavo H. Goldman

**Affiliations:** a Faculdade de Ciências Farmacêuticas de Ribeirão Preto, Universidade de São Paulo, Ribeirão Preto, Brazil; b Instituto de Química, Universidade Estadual de Campinas, Campinas, Brazil; c Departamento de Bioquímica, Instituto de Química, Universidade de São Paulo, São Paulo, Brazil; Duke University Medical Center

**Keywords:** *Aspergillus fumigatus*, *Pseudomonas aeruginosa*, biofilm formation, gliotoxin, hypoxia and normoxia, secondary metabolites

## Abstract

In cystic fibrosis (CF), mucus plaques are formed in the patient’s lungs, creating a hypoxic condition and a propitious environment for colonization and persistence of many microorganisms. There is clinical evidence showing that Aspergillus fumigatus can cocolonize CF patients with Pseudomonas aeruginosa, which has been associated with lung function decline. P. aeruginosa produces several compounds with inhibitory and antibiofilm effects against A. fumigatus *in vitro*; however, little is known about the fungal compounds produced in counterattack. Here, we annotated fungal and bacterial secondary metabolites (SM) produced in mixed biofilms under normoxia and hypoxia conditions. We detected nine SM produced by P. aeruginosa. Phenazines and different analogs of pyoverdin were the main compounds produced by P. aeruginosa, and their secretion levels were increased by the fungal presence. The roles of the two operons responsible for phenazine production (*phzA1* and *phzA2*) were also investigated, and mutants lacking one of those operons were able to produce partial sets of phenazines. We detected a total of 20 SM secreted by A. fumigatus either in monoculture or in coculture with P. aeruginosa. All these compounds were secreted during biofilm formation in either normoxia or hypoxia. However, only eight compounds (demethoxyfumitremorgin C, fumitremorgin, ferrichrome, ferricrocin, triacetylfusigen, gliotoxin, gliotoxin E, and pyripyropene A) were detected during biofilm formation by the coculture of A. fumigatus and P. aeruginosa under normoxia and hypoxia conditions. Overall, we showed how diverse SM secretion is during A. fumigatus and P. aeruginosa mixed culture and how this can affect biofilm formation in normoxia and hypoxia.

## INTRODUCTION

Pseudomonas aeruginosa is a Gram-negative bacterium that grows aerobically and under anaerobic conditions in certain specific circumstances. The species is ubiquitous in nature and has been found inhabiting soil and water and also colonizing humans, where it sometimes acts as an opportunistic pathogen (1). In immunosuppressed, burned, and hospitalized patients, P. aeruginosa is responsible for a broad spectrum of serious diseases ranging from acute to chronic infections, such as bloodstream infections in intensive care units, surgical site infections, hospital-acquired pneumonia, respiratory and urinary tract infections, and burn and chronic dermal wound infections (1, 2). P. aeruginosa also chronically infects the lungs of people with underlying pulmonary diseases, such as cystic fibrosis (CF).

CF is a genetic disorder caused by mutations in the CF transmembrane conductance regulator gene that result in defective chloride secretion, altered airway surface liquid, ciliary dyskinesis, and impaired mucociliary clearance (3). Such changes lead to motionless mucus plaques, which create a hypoxic condition and a propitious environment for colonization and persistence of many microorganisms, notably P. aeruginosa (1, 4). By performing an *in vivo* characterization of CF airways, Worlitzsch and colleagues (4) demonstrated that P. aeruginosa forms biofilm-like macrocolonies in the intraluminal site, which is markedly hypoxic due to mucus accumulation. In response to hypoxia, P. aeruginosa increases alginate exopolysaccharide production, and that may help the bacteria grow as a biofilm and persist in that environment.

In addition to P. aeruginosa and other bacteria, CF lungs can be colonized by fungi, with Aspergillus fumigatus the main isolated mold. There is a particular interest in this opportunistic pathogen, as A. fumigatus presence in respiratory CF samples has been associated with poorer prognosis and pulmonary function decline (5). A. fumigatus is a ubiquitous filamentous fungus encountered in soil, water, air, decomposing organic matter, and plant-based materials (6, 7), and it probably evolved in contact with water and soil bacteria such as P. aeruginosa. A. fumigatus can cause a range of illnesses that vary from chronic or hypersensitization (allergic reactions) disorders to invasive and life-threatening diseases (8). In CF patients, A. fumigatus may colonize the bronchi, which is frequently accompanied by hypersensitization (1, 9, 10), allergic bronchopulmonary aspergillosis (11), and bronchitis (12).

There is clinical evidence that A. fumigatus and P. aeruginosa cocolonize CF patients, and this is associated with lung function decline. Some reports estimate that 60% of patients with chronic P. aeruginosa infection also carry A. fumigatus (13–16). Studies investigating how A. fumigatus and P. aeruginosa affect each other *in vivo* and the outcome of this interaction for the host are limited; however, several studies have analyzed this interaction *in vitro*. Overall, P. aeruginosa has a strong inhibitory effect against A. fumigatus *in vitro* (including inhibition of biofilm formation and conidiation), due to bacteria-produced compounds (1, 17). The surfactant dirhamnolipids inhibit fungal growth by blocking β-1,3-glucan synthase (18), a key enzyme for fungal cell wall production; the quorum-sensing homoserine lactones act to suppress hyphal growth (19); the siderophore pyoverdine causes fungal iron starvation (17, 20, 21); and pyochelin and phenazines kill A. fumigatus by inducing oxidative and nitrosative stresses as well as iron starvation (17, 22).

**FIG 1:**
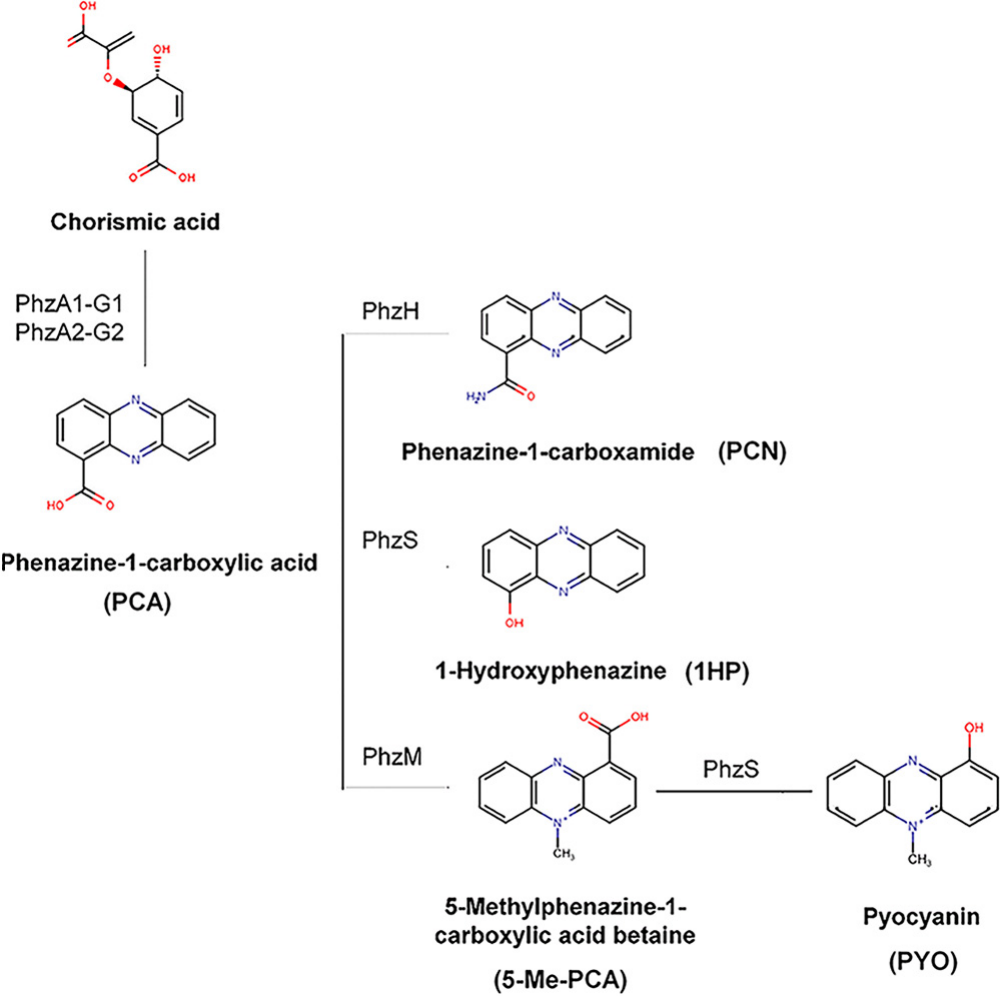
P. aeruginosa phenazine biosynthesis pathway.

Phenazines are nitrogen-containing colored aromatic molecules which constitute a large group of secondary metabolites (SM) produced by bacteria with broad physiological functions, including acting as antibiotics (2) and antifungals (22–25), involvement in biofilm formation (26), and regulation of gene expression (27). Several phenazines are produced by P. aeruginosa (Fig. 1), such as pyocyanin (PYO), phenazine-1-carboxamide (PCN), phenazine-1-carboxylic acid (PCA), 1-hydroxyphenazine (1-HP), and 5-methyl-phenazine-1-carboxylic acid (5-Me-PCA). Biosynthesis of PCA from chorismic acid requires enzymes coded by two sets of homologous genes (*phzABCDEFG*) located in two nearly identical redundant operons (*phz1* and *phz2*) that have different promoters and flanking regions (28). Another three genes, *phzM*, *phzS*, and *phzH*, code for enzymes that convert PCA to PYO and PCN and are located next to either *phz1* or *phz2* operons (28).

Although P. aeruginosa compounds with inhibitory and antibiofilm effects against A. fumigatus have been revealed, little is known about the compounds produced by the fungus during the interaction. Furthermore, most of the studies about P. aeruginosa SM during interactions were done by using mutants lacking important genes for biosynthesis of such compounds and/or by measuring the effect of adding purified compounds or bacteria-filtered supernatant to the coculture or a monoculture. There is a lack of studies identifying fungal and bacterial SM produced throughout coculturing and mixed biofilms and their roles in the fungus-bacterium interaction, especially regarding fungal metabolites.

Here, we show the SM produced by P. aeruginosa and A. fumigatus in single or mixed biofilms during normoxia and hypoxia conditions. We detected 10 SMs produced by P. aeruginosa. Phenazines and different analogs of pyoverdin were the main compounds produced by P. aeruginosa, and their secretion levels were increased by the fungal presence. The contributions of the two operons that regulate phenazine production (*phzA1* and *phzA2*) are still controversial and were also investigated. The results showed that Δ*phzA1* and Δ*phzA2* mutants can produce a subset of phenazines when in hypoxia and in the presence of the fungus. In contrast, we were able to detect 20 SM produced by A. fumigatus, but only 8 of them (demethoxyfumitremorgin C, fumitremorgin C, ferrichrome, ferricrocin, triacetylfusigen, gliotoxin, gliotoxin E, and pyripyropene A) were produced in the presence of P. aeruginosa.

## RESULTS

### A. fumigatus and P. aeruginosa biofilm formation in normoxia and hypoxia.

**FIG 2:**
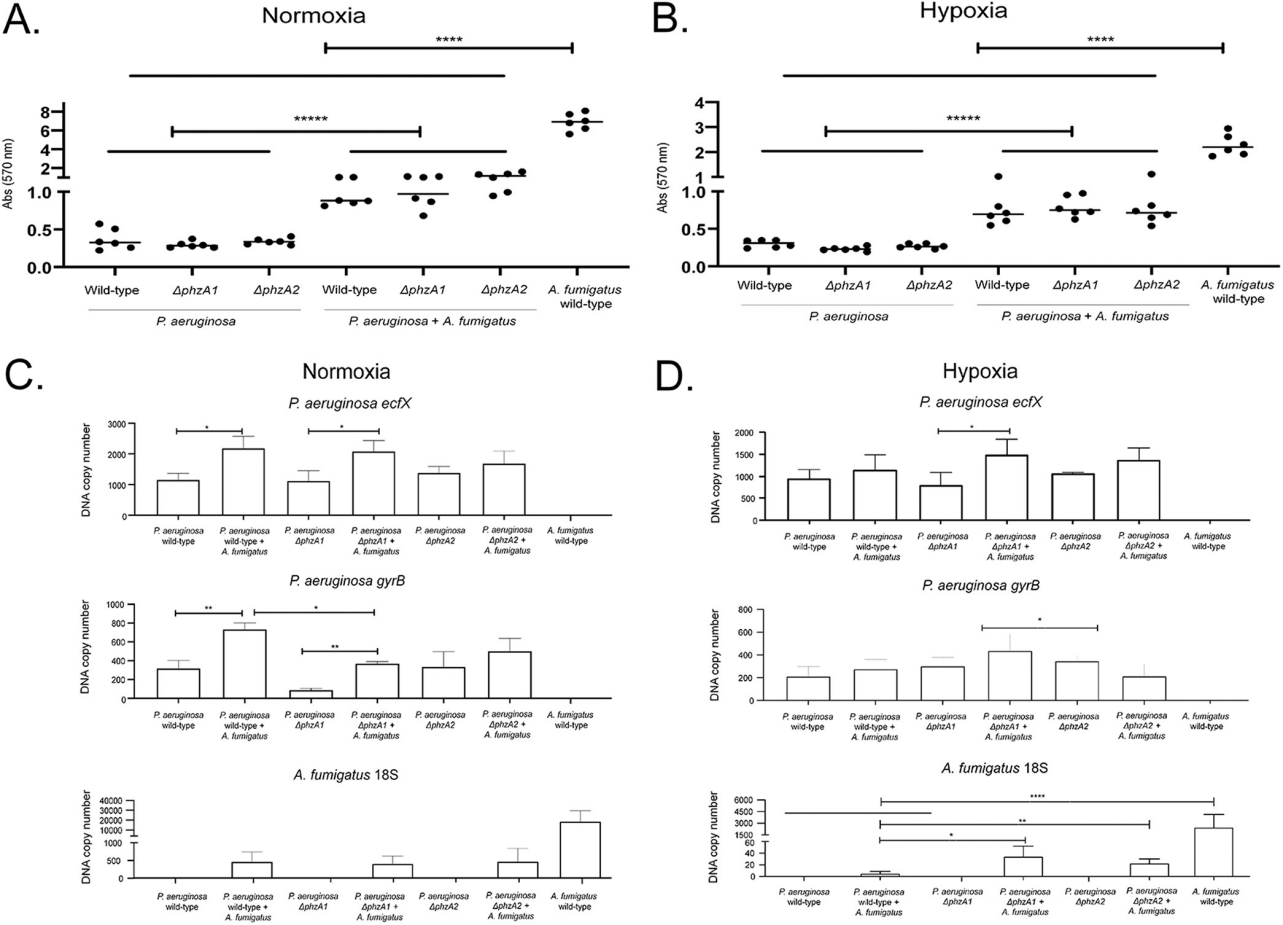
Biofilm formation by P. aeruginosa and A. fumigatus. (A and B) P. aeruginosa and A. fumigatus grown for 5 days at 37°C in normoxia and hypoxia conditions. The results of the absorbance of crystal violet are the average of six repetitions ± standard deviations. (C and D) qPCR results for P. aeruginosa *efcX* and *gyrB* and A. fumigatus 18S DNA. The results are the averages of three repetitions ± standard deviations. *, *P* < 0.05; **, *P* < 0.01; ****, *P* < 0.0001; *****, *P* < 0.00001.

We established a protocol for P. aeruginosa and A. fumigatus biofilm formation under normoxia and hypoxia by using 10^5^ CFU/mL from exponential-phase P. aeruginosa cultures and 10^6^ A. fumigatus conidia/mL (Fig. 1). P. aeruginosa wild type and Δ*phzA1* (A1) and Δ*phzA2* (A2) mutant strains were comparable in biofilm formation. Mixed biofilms of A. fumigatus-P. aeruginosa, A. fumigatus-P. aeruginosa Δ*phzA1* (AfA1), and AfA2 had more biomass than bacteria-only biofilms, in either normoxia or hypoxia (Fig. 2A and B). However, A. fumigatus biofilms without bacteria produced more biomass than mixed biofilms, indicating an antagonistic role of P. aeruginosa toward A. fumigatus biofilm formation.

These results were refined by estimating the P. aeruginosa and A. fumigatus DNA copy number by quantitative PCR (qPCR) using P. aeruginosa *ecfX* (encoding an extracytoplasmic function sigma factor unique to P. aeruginosa, also annotated as *hxuI*) and *gyrB* (encoding a DNA gyrase) and A. fumigatus 18S DNA (29–31). Corroborating the biofilm biomass results, A. fumigatus DNA copy number decreased in the presence of any P. aeruginosa strain under all conditions (Fig. 2C and D). Under normoxia conditions, we observed that P. aeruginosa wild type and A1 DNA copy number increased in the presence of A. fumigatus, but this was not true for A2, which showed the same DNA copy number with or without A. fumigatus (Fig. 2C). In contrast, under hypoxia conditions, only AfA1 showed a higher P. aeruginosa DNA copy number than A1 alone (Fig. 2D); interestingly, increased A. fumigatus DNA copy number was observed in AfA1 and AfA2 cultures compared to the A. fumigatus-P. aeruginosa wild type (Fig. 2D). This suggests that P. aeruginosa *phz* mutant strains have a lower ability to inhibit A. fumigatus biofilm than the P. aeruginosa wild-type strain.

These results strongly indicate that we have established a robust A. fumigatus-P. aeruginosa biofilm formation protocol under normoxia and hypoxia conditions.

### Secondary metabolites produced by P. aeruginosa during biofilm formation.

**FIG 3:**
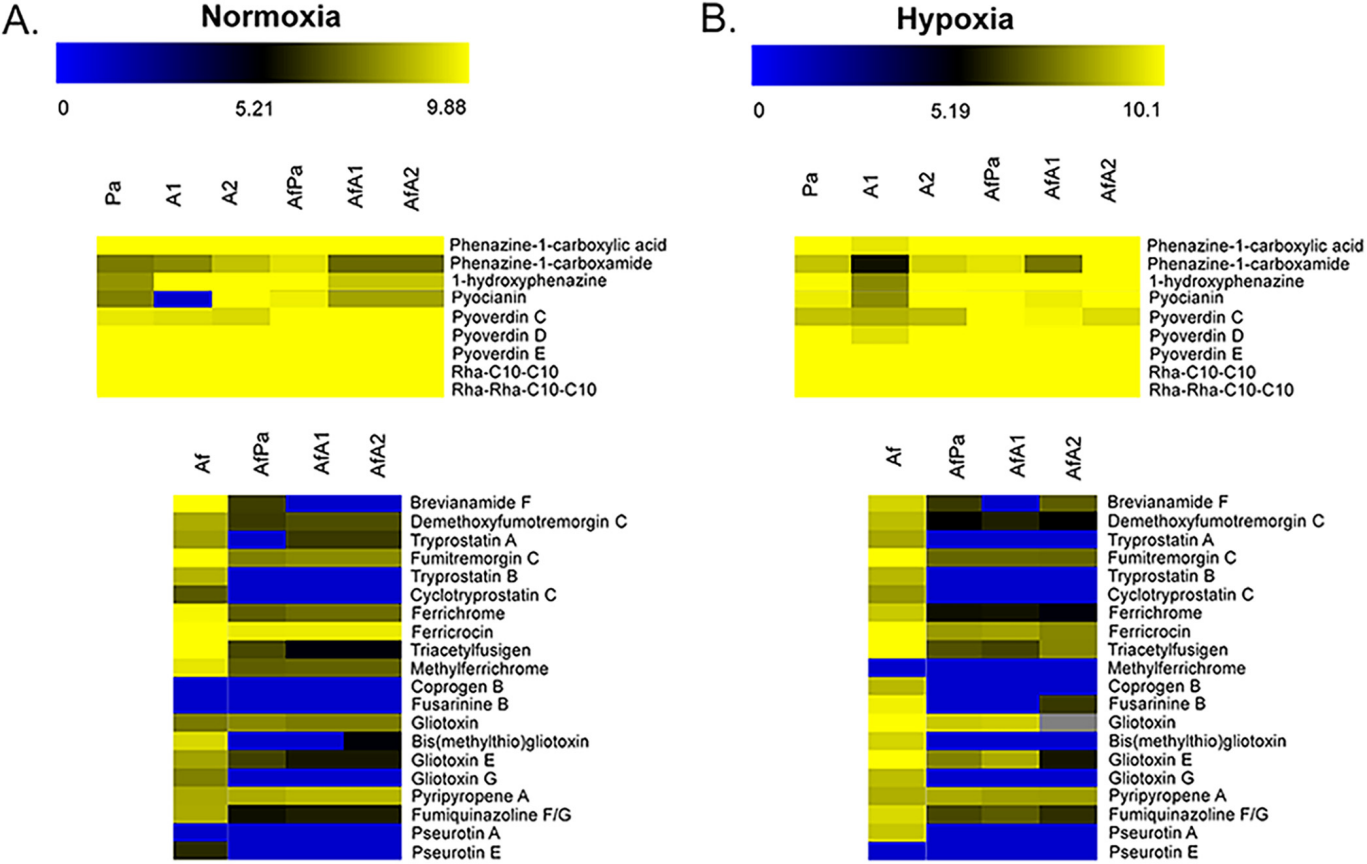
Specialized secondary metabolite production by P. aeruginosa and A. fumigatus. Heat maps depict the log_10_ of the area of chromatograms of 29 SM (9 from P. aeruginosa and 20 from A. fumigatus). The values represent the averages of three independent biological repetitions. Pa, P. aeruginosa; Af, A. fumigatus.

**FIG 4:**
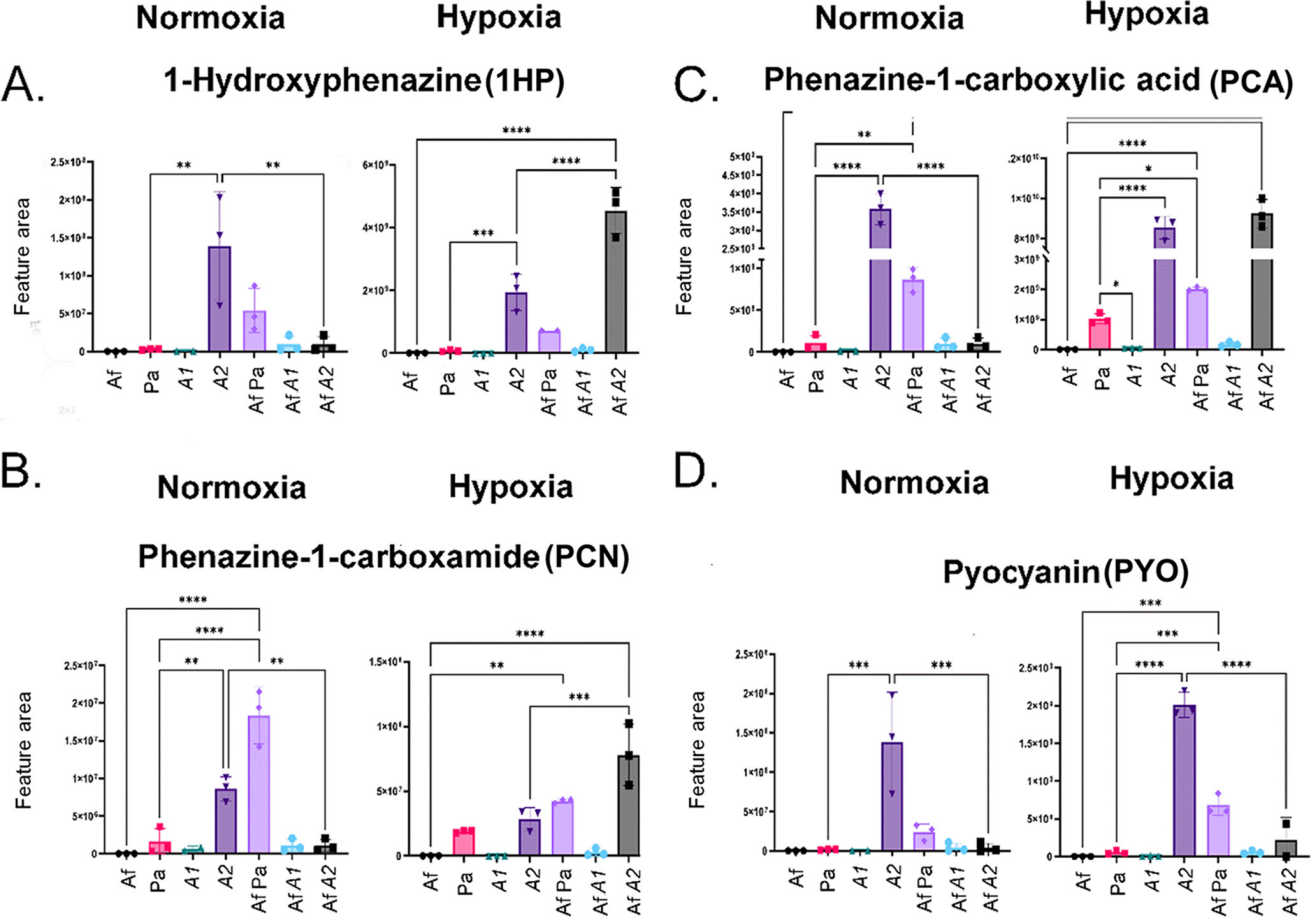
P. aeruginosa phenazine production during biofilm formation in normoxia and hypoxia. Areas of the chromatograms of 1-hydroxyphenazine (A), phenazine-1-carboxylic acid (B), phenazine-1-carboxamide (C), and pyocyanin (D) are shown. The results are the averages of three repetitions ± standard deviations. *, *P* < 0.05; **, *P* < 0.01; ***, *P* < 0.001; ****, *P* < 0.0001.

Next, we used high-performance liquid chromatography (HPLC)–high-resolution tandem mass spectrometry (HRMS^2^) to identify SM in the A. fumigatus and P. aeruginosa biofilm supernatants. We were able to annotate a total of 29 SM, 9 from P. aeruginosa and 20 from A. fumigatus, in the supernatants produced under either condition (Fig. 3; see also our supplementary data available via figshare, including Table S1 and Fig. S1 to S8, at https://doi.org/10.6084/m9.figshare.19620702). PhZA1-G1 and PhZA2-G2 pathways use chorismic acid as the precursor for transformation into PCA (Fig. 1). PCA is converted into phenazine-1-carboxamide, 1-hydroxyphenazine, and 5-methylphenazine-1-carboxylic acid betaine by PhzH, PhzS, and PhzM, respectively (Fig. 1). Subsequently, 5-methylphenazine-1-carboxylic acid betaine is converted into pyocyanin by PhzS (Fig. 1). As expected, methylphenazine-1-carboxylic acid betaine and pyocyanin were not detected in the Δ*phzA1* mutant (A1 biofilm) under normoxia conditions (Fig. 3 and Fig. 4C and E). All these compounds, except for methylphenazine-1-carboxylic acid betaine, were induced in the mixed A. fumigatus-P. aeruginosa biofilm under normoxia and hypoxia conditions, compared to a P. aeruginosa-only biofilm (Fig. 3 and Fig. 4A to E). Interestingly, the production of phenazines in A1 and A2 biofilms varied compared to those in the wild-type P. aeruginosa biofilm: whereas A1 presented low phenazine production under all conditions, A2 had higher phenazine production than wild-type P. aeruginosa or A1.

When phenazine production by AfA1 mixed biofilms was analyzed, there was an increase for all compounds under both normoxia and hypoxia conditions, compared to the A1-only biofilm; however, AfA2 biofilms only increased phenazine production under hypoxia, compared to A2-only biofilms, except for PYO. Phenazine-1-carboxamide production was comparable in the P. aeruginosa wild-type and A1 strains under hypoxia conditions (Fig. 3 and Fig. 4A to E). Phenazine production in AfA1 was much lower than that of A. fumigatus-P. aeruginosa wild type under either condition (Fig. 3 and Fig. 4A to E). As expected, phenazine production was significantly higher during biofilm formation in hypoxia than normoxia (Fig. 3 and Fig. 4A to F), as PYO can be used as an alternative electron acceptor in anaerobiosis (32).

**FIG 5:**
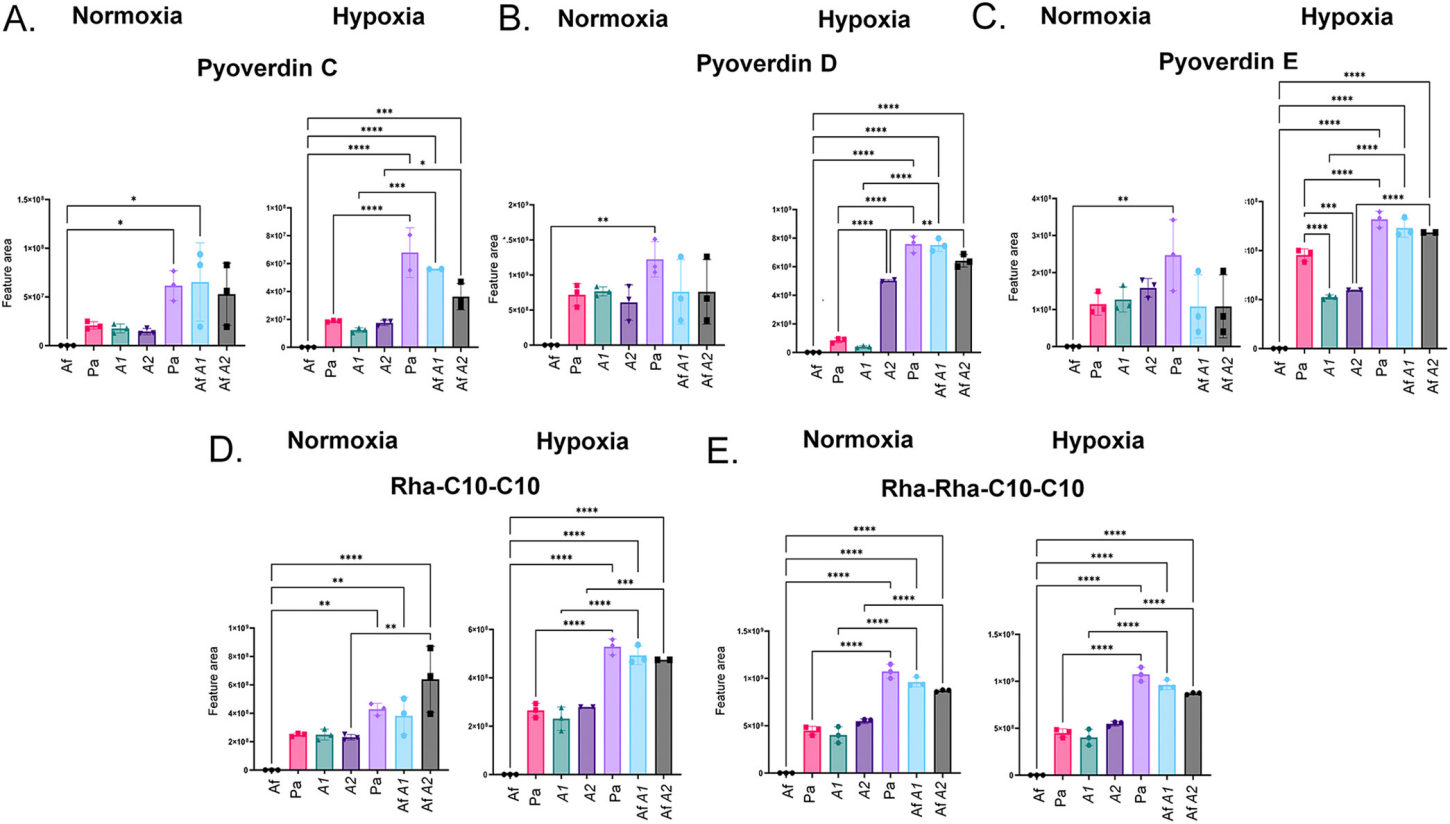
P. aeruginosa pyoverdin and rhamnolipid production during biofilm formation in normoxia and hypoxia. Areas of the chromatograms of pyoverdin C (A), pyoverdin D (B), pyoverdin E (C), Rha-C_10_-C_10_ (D), and Rha-Rha-C_10_-C_10_ (E) are shown. The results are the averages of three repetitions ± standard deviations. *, *P* < 0.05; **, *P* < 0.01; ***, *P* < 0.001; ****, *P* < 0.0001.

The siderophores pyoverdine C, D, and E and rhamnolipids Rha-C_10_-C_10_ and Rha-Rha-C_10_-C_10_ are produced in comparable amounts during biofilm formation by A. fumigatus-P. aeruginosa wild type, A1, and A2 under normoxia and hypoxia conditions (Fig. 3 and Fig. 5). The Δ*phzA1* and Δ*phzA2* mutations affected pyoverdin D and E levels during biofilm formation in hypoxia (Fig. 3 and Fig. 5B and C). All these compounds were produced in larger amounts in mixed biofilms (A. fumigatus-P. aeruginosa wild type, AfA1, and AfA2) in both normoxia and hypoxia, except for pyoverdine E under normoxia (Fig. 3 and Fig. 5A to E).

These results indicated that interaction with A. fumigatus in mixed biofilms stimulates the production of phenazines by the P. aeruginosa wild-type strain and that mutation in *phzA1* or *phzA2* modulates negatively or positively phenazine production, respectively. Pyoverdine and rhamnolipid production are not significantly different among P. aeruginosa wild type, A1, and A2, but increased production was detected in all mixed biofilms (A. fumigatus-P. aeruginosa wild type, AfA1, and AfA2).

### A. fumigatus biofilm formation induces the production of metabolites in the superpathway of fumitremorgin biosynthesis.

**FIG 6:**
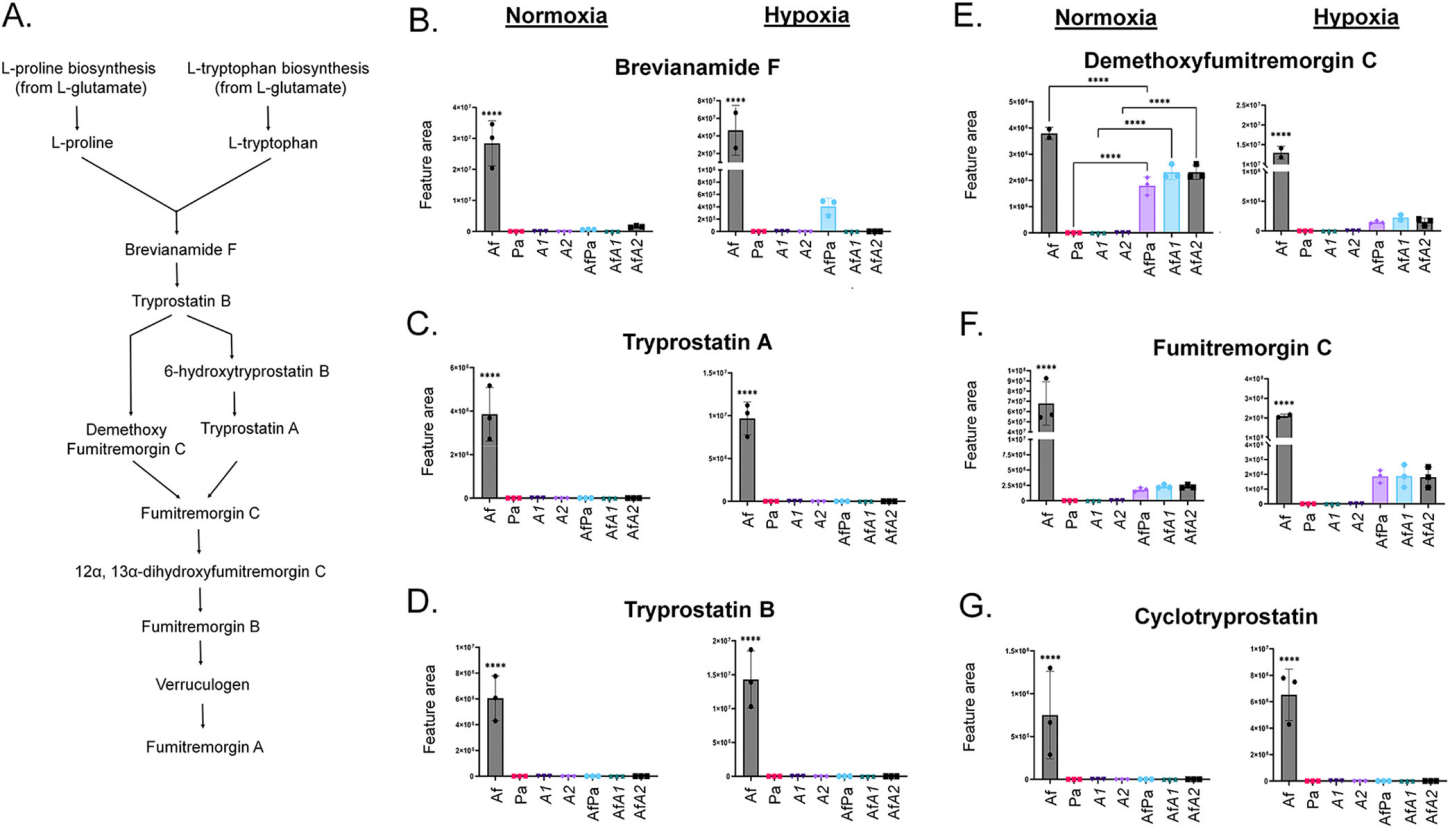
A. fumigatus biofilm formation induces the production of metabolites in the superpathway of fumitremorgin biosynthesis. (A) Superpathway of fumitremorgin biosynthesis. (B to G) Areas of the chromatograms of brevianamide F (B), tryprostatin A (C), tryprostatin B (D), demethoxyfumitremorgin C (E), fumitremorgin C (F), and cyclotryprostatin (G). The results are the averages of three repetitions ± standard deviations. ****, *P* < 0.0001.

The tremorgenic mycotoxins in the group fumitremorgins are prenylated indole alkaloids produced by A. fumigatus (33). Fumitremorgin C is produced through a series of steps in the superpathway of fumitremorgin biosynthesis (http://vm-trypanocyc.toulouse.inra.fr/META/NEW-IMAGE?type=PATHWAY&object=PWY-7525&orgids=LEISH) (Fig. 6A). Several metabolites in this pathway are produced during biofilm formation under normoxia and hypoxia conditions, such as brevianamide F, demethoxyfumitremorgin C, tryprostatin A, fumitremorgin C, tryprostatin B, and cyclotryprostatin (Fig. 3 and Fig. 6B to G). Although with lower production than those produced by A. fumigatus, the A. fumigatus-P. aeruginosa biofilm showed production of brevianamide F (in hypoxia), demethoxyfumitremorgin C (in normoxia and hypoxia), and fumitremorgin C (in normoxia and hypoxia); there were no differences between A. fumigatus-P. aeruginosa wild type, AfA1, and Af2 (Fig. 3 and Fig. 6B, E, and F). These results indicated that A. fumigatus is able to produce several metabolites in the superpathway of fumitremorgin biosynthesis during biofilm formation and in the presence of P. aeruginosa.

### There is increased production of A. fumigatus metabolites important for iron metabolism during biofilm formation.

**FIG 7:**
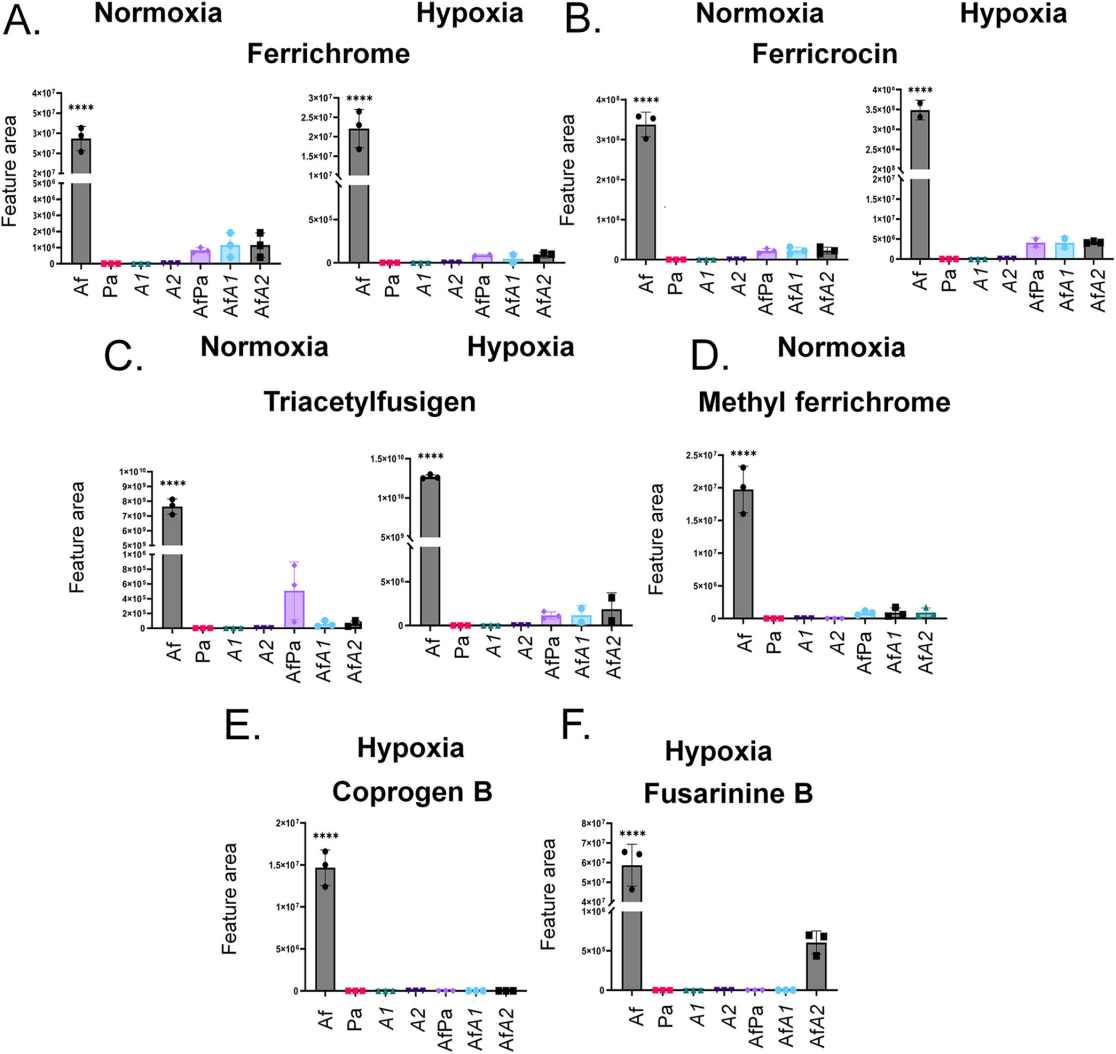
There is increased production of A. fumigatus metabolites important for iron metabolism during biofilm formation. Areas of the chromatograms of ferrichrome (A), ferrichrocin (B), triacetylfusigen (C), methylferrichrome (D), coprogen B (E), and fusarinine B (F) are shown. The results are the averages of three repetitions ± standard deviations. ****, *P* < 0.0001.

We annotated several metabolites relevant for iron assimilation, such as ferrichrome, ferricrocin, and triacetylfusigen, produced during A. fumigatus biofilm formation under normoxia and hypoxia conditions (Fig. 3 and Fig. 7A to C). Methyl ferrichrome was produced by A. fumigatus in biofilms only under normoxia (Fig. 3 and Fig. 7D), while coprogen B and fusarinine B were produced only under hypoxia conditions (Fig. 3 and Fig. 7E and F). Ferrichrome, ferricrocin, and triacetylfusigen were produced during biofilm formation by the mixed fungus-bacteria culture under both normoxia and hypoxia conditions, although at levels 10- to 1,000-fold lower than in A. fumigatus-only biofilms (Fig. 3 and Fig. 7A, B, and C). P. aeruginosa wild type and the mutant strains (A1 and A2) behaved in similar patterns regarding the production of those compounds, with a few exceptions: AfA1 biofilms did not produce ferrichrome in hypoxia or triacetylfusigen in normoxia.

Taken together, these results strongly indicate that A. fumigatus can still produce several metabolites important for iron chelation during biofilm formation in both normoxia and hypoxia conditions, notably, ferrichrome, ferricrocin, and triacetylfusigen. Nevertheless, the results also showed that P. aeruginosa strongly affects the overall production of all iron chelators detected in this approach, suggesting that competition for this micronutrient is a key point in the A. fumigatus-P. aeruginosa interaction in biofilms.

### Gliotoxin, pyripyropene A, and fumiquinazoline F and G are produced by A. fumigatus during biofilm formation.

**FIG 8:**
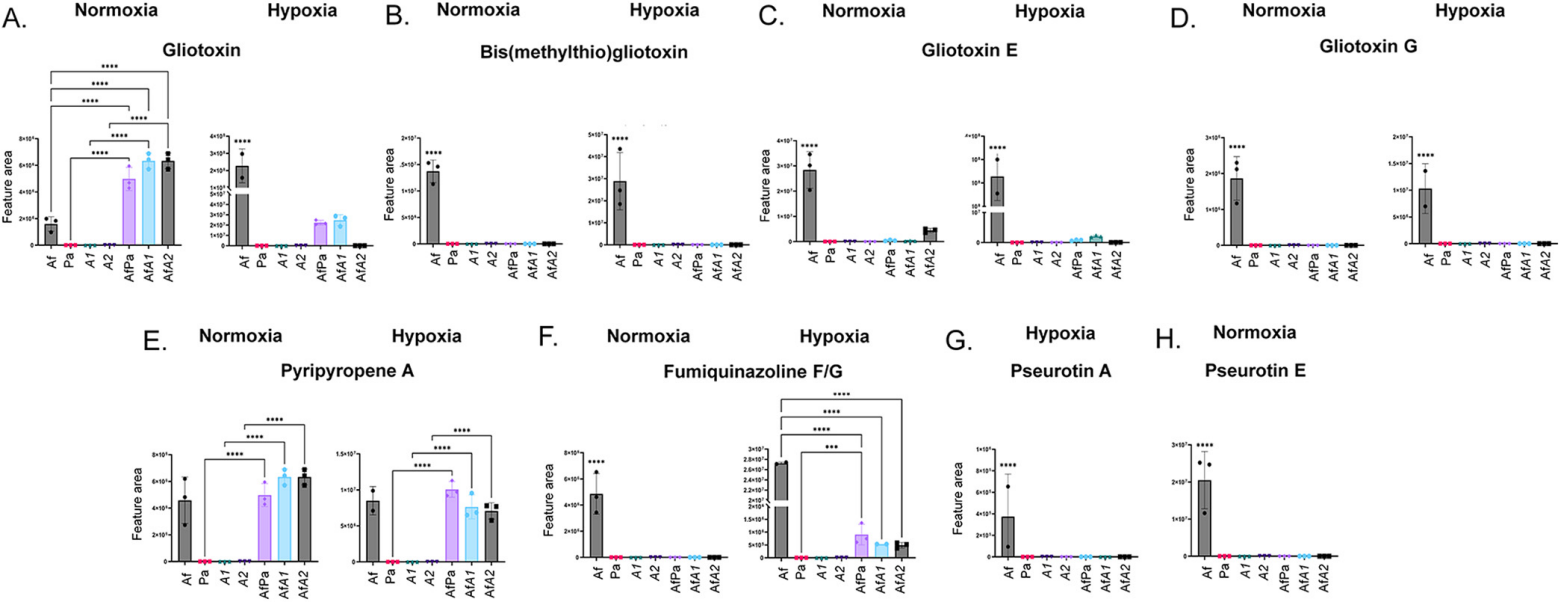
Gliotoxin, pyripyropene A, and fumiquinazoline F and G are produced during A. fumigatus biofilm formation. Areas of the chromatograms of gliotoxin (A), bisdethiobis(methylthio)-gliotoxin (B), gliotoxin E (C), gliotoxin G (D), pyripiropene A (E), fumiquinazoline F and G (F), pseurotin A (G), and pseurotin E (H) are shown. The results are the averages of three repetitions ± standard deviations. ***, *P* < 0.001; ****, *P* < 0.0001.

Gliotoxin (GT) and GT-modified forms, such as bis(methylthio) GT, GT E, and GT G, are produced during A. fumigatus biofilm formation under normoxia and hypoxia conditions (Fig. 3 and Fig. 8A to D). GT production was induced 2- to 3-fold induced in the A. fumigatus-P. aeruginosa wild type, AfA1, and AfA2 cultures during biofilm formation under normoxia conditions (Fig. 3 and Fig. 8A). Curiously, under hypoxia conditions, GT levels were lower than the A. fumigatus-only culture in the mixed biofilms, and the lack of *phzA2* function suppressed GT production completely (Fig. 3 and Fig. 8B). Pyripyropene A was produced in comparable levels in all conditions, both in A. fumigatus-only and mixed biofilms (Fig. 3 and Fig. 8E), indicating that the presence of P. aeruginosa did not influence its production. A different pattern was seen for the production of fumiquinazoline F or G in mixed biofilms, as production was completely inhibited by the bacteria in normoxia but only partially during hypoxia (Fig. 3 and Fig. 8F).

Taken together, these results emphasize the importance of GT, pyripyropene A, and fumiquinazoline F and G during A. fumigatus-P. aeruginosa biofilm formation and suggest that A. fumigatus uses GT as a defense against P. aeruginosa.

### Biofilm formation in the presence of A. fumigatus mutants impaired in SM production.

**FIG 9:**
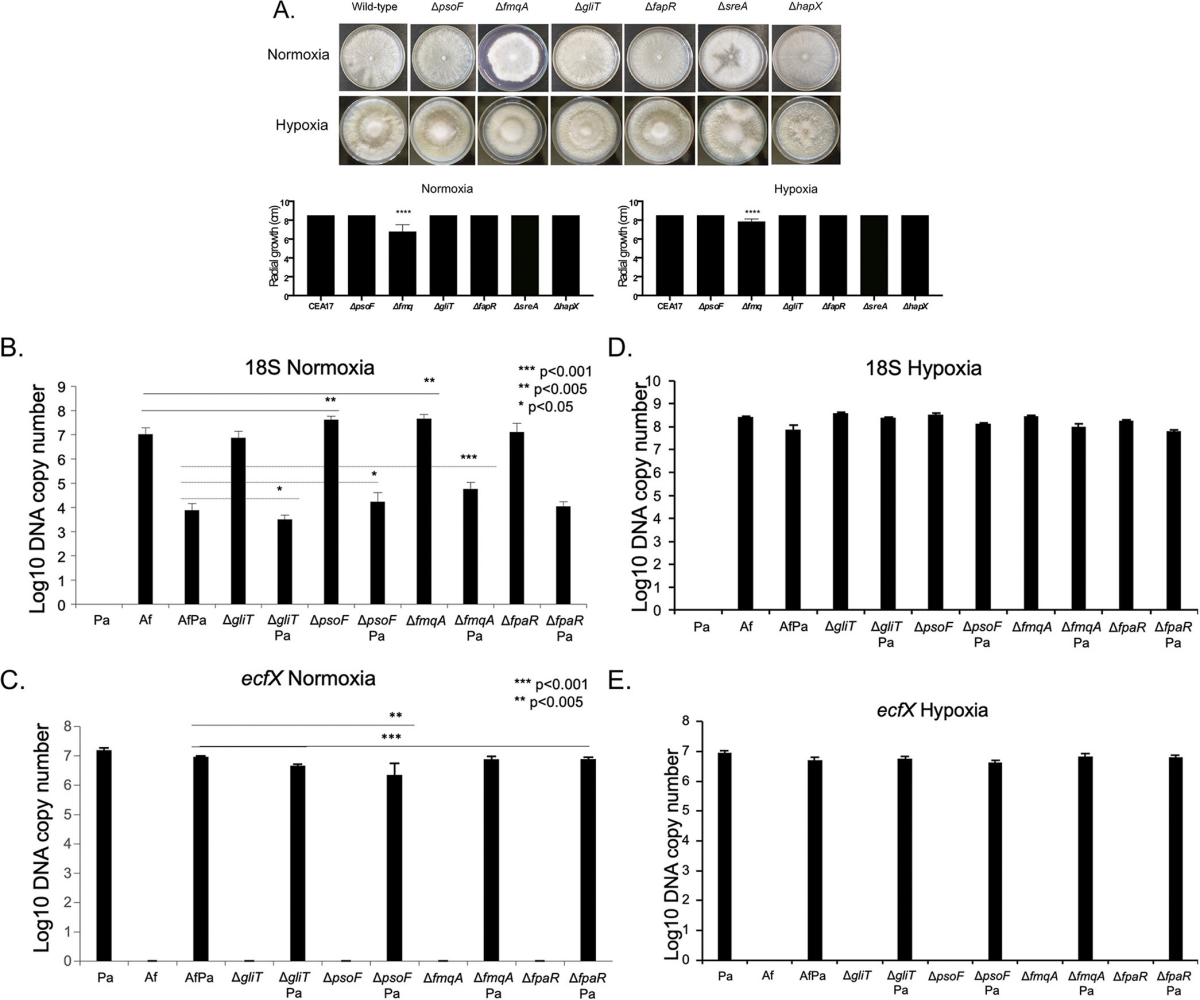
Gliotoxin is important for A. fumigatus*-*P. aeruginosa biofilm formation. (A) The wild-type and mutant strains were grown for 5 days at 37°C in normoxia and hypoxia conditions. ****, *P* < 0.0001. (B and C) qPCR for A. fumigatus 18S DNA (B) and P. aeruginosa *ecfX* (C) biofilm formation under normoxia conditions. (D and E) qPCR for A. fumigatus 18S DNA (D) and P. aeruginosa *ecfX* (E) biofilm formation under hypoxia conditions.

As a preliminary step to investigate if A. fumigatus SM are important for the establishment of A. fumigatus-P. aeruginosa biofilm formation under normoxia and hypoxia conditions, we tested several mutants impaired in the production of (i) gliotoxin (Δ*gliT*; *gliT* encodes an oxidoreductase), (ii) pseurotin (Δ*psoF*; *psoF* encodes a putative enzyme with dual function as a methyltransferase and monooxygenase), (iii) fumiquinazoline (Δ*fmqA*; *fmqA* encodes a nonribosomal peptide synthetase), and (iv) fumagillin and pseurotin (Δ*fapR*; *fapR* encodes a transcription factor) (Fig. 9). All these mutants, except for Δ*fmqA*, had radial growth on RPMI medium comparable to that of the wild-type strain in both normoxia and hypoxia conditions (Fig. 9A).

qPCR analysis showed that A. fumigatus Δ*psoF* and Δ*fmqA* had increased biofilm formation during normoxia in the absence and presence of P. aeruginosa (Fig. 9B). However, Δ*gliT* had decreased biofilm formation during normoxia only in the presence of P. aeruginosa (Fig. 9B). There were fewer P. aeruginosa cells in the biofilm dual interaction between A. fumigatus Δ*psoF* or Δ*fapR* and P. aeruginosa during normoxia conditions (Fig. 9C), while there were no statistically significant differences in the number of P. aeruginosa cells in the presence of other A. fumigatus mutants (Fig. 9C). No differences were observed between the A. fumigatus 18S wild type and mutants or with P. aeruginosa *ecfX* with A. fumigatus wild type and mutants during biofilm formation under hypoxia conditions (Fig. 9D and E).

These results suggest that gliotoxin is important for A. fumigatus-P. aeruginosa biofilm formation during normoxia conditions. Curiously, the lack of pseurotin production decreased P. aeruginosa biofilm formation during normoxia conditions, while the secondary metabolite mutants did not affect A. fumigatus-P. aeruginosa biofilm formation during hypoxia conditions.

## DISCUSSION

The clinical and scientific interests in coinfection with A. fumigatus and P. aeruginosa are due to its association with a decline in the lung function in cystic fibrosis patients, which has been shown in many reports (1). Inside the host, both pathogens have to face a hostile environment triggering general and specific responses and adapting to specific conditions and nutrient availability. Furthermore, they may interact with each other, which can boost their growth or lead to the production of antagonistic molecules. Among such molecules, some SM have been described as affecting fungal and bacterial growth and their metabolism. However, to the best of our knowledge, an overview of SM production by both microorganisms in cocultivation is missing. It is important that we have not worked with A. fumigatus and P. aeruginosa clinical isolates that chronically colonize patient lungs. A. fumigatus CEA17 is a derivative of CEA10, a clinically derived strain isolated from a patient with invasive aspergillosis (34–36). P. aeruginosa PA14, a highly virulent isolate that represents the most common clonal group worldwide, was isolated from a burn wound and not from a patient lung (37). However, we decided to work with these two clinical isolates because they have several technological resources, for instance, deletion and disruption libraries of their whole genomes, that will favor further investigation of the biological basis of their interaction. It is possible that clinical isolates of both species that are colonizing the lungs of CF patients have different SM profiles from what is described here, and this remains to be investigated. Another important observation is that the reduced production of an A. fumigatus SM does not reflect the direct inhibition of its biosynthesis by P. aeruginosa. This could be due to the effect of A. fumigatus growth inhibition by P. aeruginosa, since less fungal biomass in the mixed biofilms likely produces less metabolites.

Here, we showed that P. aeruginosa has antagonistic effects against A. fumigatus in mixed biofilms, both in normoxic and hypoxic conditions, which is in agreement with reports showing antagonistic action for bacteria isolated from clinical pulmonary samples in a normal oxygen atmosphere (23, 38) and another report that showed that P. aeruginosa inhibitory effects were effective independently of the local oxygen pressure (39). P. aeruginosa is a nonfermentative bacterium that grows anaerobically when nitrate is available, which may also be a key factor for cultivation in hypoxic conditions (39). In our work, we decided to use RPMI 1640 buffered with HEPES (pH 7.0) because it is a medium that has been used to induce biofilm formation and mimics the human plasma constitution. RPMI 1640 has calcium nitrate [Ca(NO_3_)_2_; 0.42 mM], which could support P. aeruginosa growth in hypoxia. P. aeruginosa can also survive in oxygen-limited conditions using pyocyanin as an electron acceptor to regenerate NAD^+^ (40). We performed species-specific qPCR to distinguish between A. fumigatus and P. aeruginosa in cocultured biofilms and found that A. fumigatus growth was inhibited by the presence of P. aeruginosa wild type and Δ*phzA1* and Δ*phzA2* mutants independently of the oxygen pressure. However, P. aeruginosa wild type and Δ*phzA1* showed increased growth when cocultured with A. fumigatus in normoxia. This result disagrees with the work performed by others (41) that showed a mutually antagonistic relationship between P. aeruginosa and A. fumigatus, but it confirms the data of Margalit and colleagues (42), who demonstrated that the A. fumigatus secretome could stimulate the growth of P. aeruginosa. Specifically, A. fumigatus can produce an amino acid-rich environment in which P. aeruginosa can proliferate better in cocultures (42). The increased P. aeruginosa proliferation was not observed under hypoxic conditions. We hypothesize that this occurred because the fungus does not grow well in hypoxia and possibly did not create this amino acid-rich environment that is able to boost P. aeruginosa growth. Moreover, the P. aeruginosa growth rate in hypoxia is lower than in aerobic conditions, even in the presence of nitrate and arginine, which can also be used as terminal electron acceptors (43).

The antifungal effect of P. aeruginosa-produced compounds on A. fumigatus has been extensively studied, and several molecules can interfere with fungal morphology, physiology, and growth. In our work, we annotated many phenazines, such as PYO, PCN, PCA, and 1-HP, that are produced by the bacterium in single and mixed cultures, both in hypoxia and normoxia. The antagonistic actions of phenazines are attributed to their redox potential, since reduced phenazines are oxidized in the fungal cell by oxygen and NADPH through a NapA-dependent oxidative stress response, generating reactive oxygen species (ROS) (17, 22). All phenazines at high concentrations induce ROS and reactive nitrogen species (RNS) production by A. fumigatus mitochondria, which are released into the cytoplasm and lead to fungal death (44). 1-HP is the most active phenazine against A. fumigatus and, in addition to ROS and RNS production, its high inhibitory activity is due to a specific iron chelation property (45). P. aeruginosa phenazine production is controlled by a complex regulatory network that involves quorum sensing and catabolite repression (44). Two redundant *phz* gene operons are responsible for phenazine-1-carboxylate production (46–49). Earlier work showed that in P. aeruginosa colony biofilms, *phzA2* was expressed at high levels whereas *phzA1* was the most important operon for PCA production (47). Recent analysis suggested a dominant role of *phzA2*, resulting in a 10-fold-higher expression of *phzA2* compared to *phzA1*, and *phzA2* operon as the main responsible for PCA production (49), but there are marked differences in quorum-sensing regulated traits depending on the particular P. aeruginosa strain and specific growth conditions. Surprisingly, in contrast, our results showed that PC production is higher with the Δ*phzA2* strain both in monoculture or A. fumigatus coculture than in the Δ*phzA1* mutant.

Another P. aeruginosa-produced compound that is induced upon iron starvation is pyoverdine (17), and some authors have shown that this is the main mediator of antifungal activity on A. fumigatus biofilms (17, 20). Also, there have been some reports describing that pyoverdine is produced in lower levels under iron-limiting conditions (20) or under hypoxia (39). However, our results showed that P. aeruginosa is able to produce pyoverdine in RPMI medium, which is a poor-iron medium, and in a low-oxygen atmosphere. Except for pyoverdine D, there is little influence of the Δ*phzA1* and Δ*phzA2* null mutations on the production of pyoverdine C, D, or E, as expected, since the regulation of phenazines and pyoverdine are independent.

Under our conditions, the rhamnolipids Rha-C_10_-C_10_ and Rha-Rha-C_10_-C_10_ were also detected in all P. aeruginosa strain supernatants, and growth in coculture with A. fumigatus increased their concentration (Fig. 4D and E). Rhamnolipids are surfactants released by P. aeruginosa that have several roles, such as allowing swarming motility and solubilizing hydrophobic compounds that can be used as carbon and energy sources. In host-pathogen interactions, rhamnolipids are considered virulence factors, as they may help to lyse host cell membranes, interfere with signaling pathways, and solubilize the lung surfactant. They are also toxic to other bacteria, fungi, and other microorganisms, conferring a competitive advantage in colonizing multiple environments (50). In mixed A. fumigatus-P. aeruginosa biofilms, the induction of rhamnolipid production might be one of the factors that interferes with A. fumigatus growth, but the specific role of rhamnolipids in these interactions could not be addressed in this work.

Recently, quantitative proteomic analysis showed that A. fumigatus exposed to P. aeruginosa culture filtrate had increased expression of proteins involved in SM biosynthesis, such as gliotoxin, fumagillin, and pseurotin A (51). To shed light on how A. fumigatus responds to all these compounds produced by P. aeruginosa, we also annotated and performed relative quantification of fungal SM. We detected 20 SM secreted by A. fumigatus either in monoculture or in coculture with P. aeruginosa. All these compounds were secreted during fungal biofilm formation either in normoxia or hypoxia. However, we were only able to detect and annotate eight compounds (demethoxyfumitremorgin C, fumitremorgin, ferrichrome, ferricrocin, triacetylfusigen, gliotoxin, gliotoxin E, and pyripyropene A) produced during biofilm formation by the coculture of A. fumigatus and P. aeruginosa upon either normoxia or hypoxia conditions. Interestingly, brevianamide F and fumiquinazoline F and G were produced only upon hypoxia conditions, while methyl ferrichrome was produced only upon normoxia conditions. These results indicate that these SM are important for the interaction between A. fumigatus and P. aeruginosa, and the production of some of them is regulated by the oxygen condition. Of note, gliotoxin was the only SM produced in higher levels in mixed biofilms compared to A. fumigatus-only biofilms, suggesting that the fungus specifically overproduces this compound in response to the bacterial antagonist. Gliotoxin has been the most well-studied and characterized SM from A. fumigatus, and it is also important for the interaction with P. aeruginosa and for biofilm formation (52, 53). Reece and colleagues (41) showed that gliotoxin has an antibacterial activity and antibiofilm effect against several bacteria, including P. aeruginosa. Our results emphasize the importance of gliotoxin during the interaction between A. fumigatus and P. aeruginosa, because it is the only compound produced by A. fumigatus in significantly higher amounts in mixed biofilms than by A. fumigatus-only biofilms, despite the lower fungal DNA copy number in the presence of bacteria (Fig. 7A). It would be interesting to investigate the molecular mechanisms involved in such overexpression of GT induced by P. aeruginosa. There was little influence of *phzA1* and *phzA2* mutations on the gliotoxin production in normoxia compared to wild type P. aeruginosa. However, lack of *phzA2* function upon hypoxia dramatically decreased gliotoxin production, which might correlate with higher levels of phenazines, except for PYO, under this condition (Fig. 3). Furthermore, we showed that an A. fumigatus Δ*gliT* mutant, impaired in gliotoxin production, had less growth in the presence of P. aeruginosa in normoxia conditions, emphasizing its importance for controlling bacterial growth. Further work is needed to investigate if gliotoxin indeed has a direct role in the A. fumigatus-P. aeruginosa interaction and to unravel its effects in bacterial physiology in mixed biofilms.

P. aeruginosa produces iron chelators that may cause an iron starvation environment for the fungus that results in its anti-Aspergillus effect. However, the fungus counterattacks the iron deficiency by also producing siderophores (21). Siderophores are ferric iron chelators, structurally separated into different classes named hydroxamates, catecholates, carboxylates, phenolates, and mixed class. Hydroxamates are subdivided into rhodotorulic acid-, ferrioxamine-, fusarinine-, coprogen-, and ferrichrome-type siderophores and are the ones that are produced by A. fumigatus (54). In our assay, fusarinine B, coprogen B, and ferrichrome were detected in monoculture and cocultures. The RPMI medium was an iron-deficient medium, similar to human plasma, and that is probably the reason why A. fumigatus produced many siderophores in monoculture. However, only ferrichrome, ferricrocin, and triacetylfusigen were produced by A. fumigatus in the presence of P. aeruginosa during biofilm formation in both normoxia and hypoxia. Ferrichrome and ferricrocin were produced in comparable amounts in the presence of both P. aeruginosa wild-type and mutant strains upon normoxia or hypoxia. However, triacetylfusigen was produced in larger amounts in the presence of the P. aeruginosa wild type than the mutant strains. In hypoxia conditions, fusarinine B was produced only in the presence of the P. aeruginosa Δ*phzA2* mutant, again indicating an interaction between the *phz* operons and A. fumigatus SM production. Previously, the importance of A. fumigatus siderophores for the iron competition with P. aeruginosa has been reported, and the participation of several genetic determinants (*hapX*, *sidA*, *sidF*, *sidG*, and *mirB*) involved in iron starvation adaptation in response to P. aeruginosa 1-HP has been demonstrated (17, 44, 45, 55). However, to the best of our knowledge this is the first time A. fumigatus siderophores have been directly identified during A. fumigatus-P. aeruginosa coculture biofilm formation.

A. fumigatus also produced pyripyropene in monoculture or dual cultures. Pyripyropene A was originally identified as a potent inhibitor of acyl-CoA cholesterol acyltransferase (56–58), a mammalian intracellular enzyme located in the endoplasmic reticulum that forms cholesteryl esters from cholesterol (59). Pyripyropene A also shows insecticidal activity against agricultural insect pests (58, 60). It is not known if there is any correlation between these activities and aspergillosis or in the competition with bacteria, and its identification in A. fumigatus*-* P. aeruginosa dual cultures is a completely novel observation. In our mixed biofilm settings, the bacterial target remains to be uncovered.

Other SM secreted by the fungus during the coculture interaction belong to the superpathway of fumitremorgin; these SM are prenylated indole alkaloid compounds produced by A. fumigatus and *Penicillium* spp. that can act as mycotoxins (61). All the compounds identified in the fumitremorgin superpathway are produced during A. fumigatus biofilm formation. However, only demethoxyfumitremorgin C and fumitremorgin are produced during the interaction with P. aeruginosa wild-type and mutant strains. Curiously, brevianamide F is produced only upon hypoxia in the presence of P. aeruginosa wild type but not mutant strains, which suggests that *phzA1* and *phzA2* functions are important for brevianamide F production. Demethoxyfumitremorgin C has been shown to inhibit the cell viability and induce apoptosis of PC3 human advanced prostate cancer cells (62). Fumitremorgin C has been described as an inhibitor of a multidrug resistance protein that mediates resistance to chemotherapeutics in breast cancer treatment, inhibiting the growth of several phytopathogenic fungi, showing lethality to brine shrimp, and displaying antifeedant activity toward armyworm (63–65). We also observed fumiquinazolines, which normally accumulate in A. fumigatus conidia (66) and are secreted during both monoculture and dual cultures of A. fumigatus and P. aeruginosa in hypoxia. It has been reported that fumiquinazoline F from Penicillium coryphilum has antibacterial activity against Staphylococcus aureus and Micrococcus luteus (67). It remains to be investigated if all these compounds can affect P. aeruginosa physiology and growth and their mechanisms of action, if any.

In conclusion, we have annotated several SM secreted during A. fumigatus and P. aeruginosa biofilm formation, and this has provided several opportunities to understand the interaction between these two species. Further work will concentrate on the investigation of the roles of selected compounds in both fungal and bacterial competitors, and future data may be used for the development of novel drugs for the management of chronic infections that affect cystic fibrosis patients or other immunocompromised individuals.

## MATERIALS AND METHODS

### P. aeruginosa and A. fumigatus strains and growth conditions.

The following species and strains were used in this work: P. aeruginosa UCBPP-PA14 (wild type [68]), Δ*phzA1* (PA14 *phzA1::Mr7* [69]), and Δ*phzA2* (PA14 with an in-frame deletion in *phzA2*; a gift from E. Déziel), and A. fumigatus CEA17, Δ*gliT*, Δ*psoF*, Δ*fmqA*, and Δ*fapR*. P. aeruginosa was grown from frozen stocks ([LB] medium plus 20% glycerol) in solid LB for 24h at 37°C. A single colony was transferred to 30 mL of LB and cultured overnight at 37°C, 200 rpm. The culture was centrifuged at 4,000 × *g*, for 5 min, and the pellet was washed with 10 mL of phosphate-buffered saline (PBS). After centrifugation, the pellet was resuspended in LB and the inoculum was adjusted, using a spectrophotometer, to an optical density at 600 nm (OD_600_) of 0.07 to 0.075. This inoculum was grown in 30 mL of LB at 37°C, 200 rpm, for 5 h, and the centrifugation and PBS washing processes were repeated. The final pellet was resuspended in RPMI-HEPES, and the inoculum was adjusted to an OD of 0.07 to 0.075 (approximately 5 × 10^8^ to 8 × 10^8^ CFU/mL).

A. fumigatus strains were grown from frozen stocks, and conidia suspensions were obtained by harvesting grown mycelia on minimal medium plates as described by Ries and colleagues (70).

### Dual biofilm formation between P. aeruginosa and A. fumigatus.

To measure the interaction between P. aeruginosa and A. fumigatus and to determine the SM produced by them in single or cocultures, 1 × 10^5^ CFU/mL of P. aeruginosa were inoculated with or without 1 × 10^6^ conidia/mL of A. fumigatus in 15 mL of RPMI-HEPES medium into polystyrene Petri dishes (60 × 15 mm) under hypoxic (1% O_2_, 5% CO_2_) or normoxic (approximately 20% O_2_ and 0.04% CO_2_) conditions, at 37°C. After 5 days, the supernatant was collected, the plate was washed with 10 mL of ultrapure water to collect the cells, and both were transferred to a 50-mL tube. This mixture was centrifuged at 4,000 × *g* for 15 min at 4°C to obtain the pellet, which was used for qPCR assays, and the supernatant (20 mL), which was filtered through a 0.22-μm filter, was frozen and lyophilized for SM extraction.

On the bottom of the small Petri dishes used in this experiment, biofilm production was measured by the crystal violet (CV) method. The biofilm was dried at 37°C for 30 min and then stained with 5 mL of 0.05% (wt/vol) CV for 10 min. The plates were washed with 50 mL of PBS, and the CV was solubilized with 3 mL of 95% ethanol. Samples of 100 μL were transferred to 96-well plates, and the absorbance at 595 nm was determined, as a measure for biofilm formation.

### Dual quantification of species-specific biofilm growth by qPCR.

For DNA extraction, pellets obtained from cultures for evaluating the P. aeruginosa-A. fumigatus interaction were frozen and lyophilized before being triturated by adding 2-mm glass beads and 0.1-mm zirconia-silica beads and vortexing for 5 min. To the resulting powder, 1 mL of extraction buffer was added, and the tubes were vortexed for 5 min. Tubes were incubated in a water bath at 70°C for 45 min; every 10 to 15 min, the tubes were removed from the water bath and vortexed for 5 min. One milliliter of phenol-chloroform (1:1) was added to the mixture, and tubes were vortexed for 5 min. The content was transferred to 2-mL tubes and centrifuged at 14,000 × *g* for 15 min at room temperature. The supernatants were collected and transferred to 1.5-mL tubes, and 600 μL of isopropanol (Merck) was added. Samples were incubated at 4°C for 1 h before being centrifuged at 14,000 × *g* for 15 min at 4°C. The supernatant was discarded, the pellet was washed with 200 μL of 70% ethanol, and air dried for 15 min at room temperature, after which the pellet was resuspended in deionized water and treated with RNase (Promega).

P. aeruginosa DNA was specifically quantified by qPCR with primers for *ecfX* (ecfX-F, 5′-CGCATGCCTATCAGGCGTT-3′, and ecfX-R, 5′-GAACTGCCCAGGTGCTTGC-3′) and *gyrB* (gyrB-F, 5′-CCTGACCATCCGTCGCCACAAC-3′, and gyrB-R, 5′-CGCAGCAGGATGCCGACGCC-3′) (29, 30). A. fumigatus was quantified by amplification of 18S rDNA (18S fw, 5′-GACCTCGGCCCTTAAATAGC-3′, and 18S rv, 5′-CTCGGCCAAGGTGATGTACT-3′). The 10-μL qPCR mixture was composed of 5 μL SYBR green PCR master mix (Applied Biosystems, Foster City, CA, USA), 2.5 pmol of each primer, and 100 ng DNA. Cycling was performed on the ABI 7500 fast real-time PCR system with an initial hold at 95°C for 15 min, followed by 45 cycles at 95°C for 15 s and 60°C for 1 min, with a cycle threshold of 35. Negative controls without DNA were included in each qPCR run.

### SM extraction and UHPLC-HRMS^2^ analysis.

SM were extracted from 50 mg freeze-dried sample of the entire supernatant of each sample by resuspension in 1 mL of HPLC-grade methanol (MeOH), followed by 1 h of sonication in an ultrasonic bath. For sample preparation, 500 μL of each obtained extract was filtered (0.22-μm filter), transferred to vials, and diluted with HPLC-grade MeOH to a total volume of 1 mL.

UHPLC-HRMS^2^ positive-mode analysis was performed in a Thermo Scientific QExactive hybrid Quadrupole-Orbitrap mass spectrometer coupled to a Dionex UltiMate 3000 RSLCnano UHPLC system. For the stationary phase, a Thermo Scientific column, Accucore C_18_ 2.6 μm (2.1 mm by 100 mm) was used. The mobile phase was 0.1% formic acid (A) and acetonitrile plus 0.1% formic acid (B). Eluent profiles (A/B percentages) were 95/5 up to 2/98 within 10 min, maintaining 2/98 for 5 min, down to 95/5 within 1.2 min, and maintaining for 8.8 min. Total run time was 20 min for each run, and flow rate was 0.3 mL · min^−1^. The injection volume was 5 μL. MS spectra were acquired with *m/z* ranges from 100 to 1,500, with 70,000 for mass resolution. Ionization parameters were a sheath gas flow rate of 45 L.h^−1^, auxiliary gas flow rate of 10 L.h^−1^, sweep gas flow rate of 2 L.h^−1^, spray voltage of 3.5 kV, capillary temperature of 250°C, S-lens RF level of 50, and auxiliary gas heater temperature of 400°C. MS^2^ spectra were acquired in data-dependent acquisition mode. Normalized collision energy was applied stepwise (20, 30, and 40) V, and the 5 most intense precursors per cycle were measured with 17,500 resolution.

### UHPLC-HRMS^2^ data processing and FBMN.

Raw UHPLC-HRMS^2^ data were converted into mzXML format files using MSConvert (71), with 32-bit binary encoding precision, zlib compression, and peak peaking. Feature detection was performed in MZmine2 (v.2.53) (72). For MS^1^ spectra mass detection, an intensity threshold of 1E5 was used, and for MS^2^ an intensity threshold of 1E3 was used. For MS^1^ chromatogram building (73), a 5-ppm mass accuracy and a minimum peak intensity of 5E5 was set. Extracted ion chromatograms (XICs) were deconvolved using the baseline cut-off algorithm at an intensity of 1E5, minimum peak height of 3E5, and a peak duration range from 0.05 to 2 min. After chromatographic deconvolution, XICs were matched to MS^2^ spectra within *m/z* 0.02 and 0.2-min retention time windows. Isotope peaks were grouped with 5-ppm mass tolerance, 0.1-min retention time tolerance, and a maximum charge of 2. Detected peaks in different samples were aligned with a 5-ppm tolerance, 75% weight for *m/z* determinations, and 25% for retention time. MS^1^ features without MS^2^ features assigned were filtered out of the resulting matrix as well as features that did not contain isotope peaks and that did not occur in at least three samples. Finally, the feature table was exported as a .csv file, and corresponding MS^2^ spectra were exported as .mgf files. Features observed in blank samples were filtered.

A molecular network was created with the feature-based molecular networking (FBMN) workflow (74) on GNPS (https://gnps.ucsd.edu) (75). The data were filtered by removing all MS^2^ fragment ions within 17 Da of the precursor *m/z*. MS^2^ spectra were window filtered by choosing only the top 6 fragment ions in the ±50-Da window throughout the spectrum. The precursor ion mass tolerance was set to 0.02 Da, and the MS^2^ fragment ion tolerance was set to 0.02 Da. A molecular network was then created in which edges were filtered to have a cosine score above 0.65 and more than 4 matched peaks. Furthermore, edges between two nodes were kept in the network if and only if each of the nodes appeared in each other’s respective top 10 most similar nodes. Finally, the maximum size of a molecular family was set to 100, and the lowest-scoring edges were removed from molecular families until the molecular family size was below this threshold. The spectra in the network were then searched against GNPS spectral libraries (75, 76). The library spectra were filtered in the same manner as the input data. All matches kept between network spectra and library spectra were required to have a score above 0.65 and at least 4 matched peaks. Dereplicator Plus was used to annotate MS/MS spectra (77). The molecular networks were visualized using Cytoscape software (78). Resulting networks were displayed and analyzed with Cytoscape (v.3.8.2).

### Metabolite annotation.

For SM dereplication, metabolites were annotated based on the GNPS MS^2^ database via the FBMN and Dereplicator Plus workflows available on the GNPS platform. Other metabolites were manually searched against natural products databases such as the Dictionary of Natural Products, and the acquired MS^2^ spectra were compared to spectra deposited either on the GNPS database or previously published in the literature. The compounds gliotoxin, gliotoxin E, pseurotin E, and spirotryprostatin A were annotated based on their exact masses.

### Data availability.

The molecular networking job can be publicly accessed at https://gnps.ucsd.edu/ProteoSAFe/status.jsp?task=0b027bfab9064518945341d3c24ba2a9, and the Dereplicator Plus job can be accessed at https://gnps.ucsd.edu/ProteoSAFe/status.jsp?task=415264520a41492a9b43b47e623028ed for hypoxia conditions and at https://gnps.ucsd.edu/ProteoSAFe/status.jsp?task=08a9ebb5609745d5a6f805896e527b67 and https://gnps.ucsd.edu/ProteoSAFe/status.jsp?task=e59e11f719cf4ae5beb406090a987f01 for normoxia.
